# GnRH agonist trigger versus hCG trigger in GnRH antagonist in IVF/ICSI cycles: A review article

**Published:** 2016-09

**Authors:** Ashraf Alyasin, Shayesteh Mehdinejadiani, Marzieh Ghasemi

**Affiliations:** 1 *Department of Endocrinology and Infertility, Shariati Hospital, School of Medicine, Tehran University of Medical Sciences, Tehran, Iran.*; 2 *Department of Anatomy, School of Medicine, Tehran University of Medical Science, Tehran, Iran.*; 3 *Pregnancy Health Research Center, Department of Obstetrics and Gynecology, School of Medicine, Zahedan University of Medical Sciences, Zahedan, Iran.*

**Keywords:** *Gonadotropin-releasing hormone*, *Human chorionic gonadotropin*, *IVF/ICSI cycles*

## Abstract

Routinely, a bolus of 5.000-10.000 IU human chorionic gonadotropin (hCG) is used for the final follicular maturation and ovulation as a standard method. HCG has the same effect of luteinizing hormone (LH) with long half-life. It has the long lutheotrophic effect which increases the risk of ovarian hyper stimulation syndrome (OHSS). Recently, gonadotropin-releasing hormone agonist (GnRH-a) trigger has been used for the induction of final follicular maturation and ovulation with the aim of reducing the OHSS risk. Several studies have shown that the releases of endogenous follicular stimulating hormone (FSH) and LH after administration of GnRH agonist in in vitro fertilization (IVF) cycles are able to precede the final follicular maturation leading to removal of fertile oocyte with normal development of the embryo and ultimately pregnancy. But based on the results of some studies, using GnRH-a trigger leads to defect luteal-phase resulting to reduce the implantation and clinical pregnancy rates and also increase abortion in fresh embryo transfer cycles compared to routine IVF cycle with hCG triggering . Also, in recent years, studies have continued to modify the luteal phase support, so that the fresh embryo transfer is possible too. In this review, we examined the benefits, problems, and also ways to reform GnRH agonist triggering complications.

## Introduction

Gonadotropin-releasing hormone agonist (GnRH) is secreted from the mediobasal of the hypothalamus in the follicular phase of the menstrual cycle in a periodic pulse and is discharged into the pituitary portal system and bound to its receptors on gonadotroph cells in the anterior pituitary. Following, low and pulse release of follicular stimulating hormone (FSH) and luteinizing hormone (LH) happens which is necessary for the follicular growth and the ovarian secretion of estrogen. In the mid-cycle, in the presence of high levels of estrogen and low increased levels of progesterone, sudden surge of gonadotropins especially LH takes place which induces ovulation after 36-40 hrs (1).

Assisted reproductive technology (ART) consisting in vitro fertilization (IVF), intracytoplasmic sperm injection (ICSI) and intrauterine insemination (IUI) are based on the exact timing of ovulation, oocyte pick-up before ovulation and then insemination of oocyte (2). Due to biological activity of human chorionic gonadotropin (hCG) similar to LH, since the mid-1970s exogenous hCG has been used to trigger the final oocyte maturation. The release of oocyte occurs usually 36-40 hours after induction of ovulation similar to natural ovulation (2, 3).

Stimulation of the gonadotropin surge for the final oocyte maturation in the midcycle was investigated in the 1970s and then by several research groups in the 1990s. As early as 1973, in Japan, Nakano et al. illustrated that ovulation in human could be induced by infusion of 600 µg GnRH synthetic for 6 hours and then followed by single dose 400 µg subcutaneously (4). However, some researchers have suggested that GnRH antagonist cycles may increase the hypophysis sensitivity in response to GnRH-a triggering (5). 

In various studies using a dose or more GnRH agonist was proposed in the mid-cycle for gonadotropin surge stimulation. In this way it was observed that release of both gonadotropins, LH/FSH, was similar to natural condition; as well shorter duration of increasing LH avoids incidence of ovarian hyperstimulation syndrome (OHSS) (6). In this review, based on existing studies on GnRH-a triggering, the advantages, problems, and also ways to reform its complications would be addressed.

**The advantages of using GnRH-a in the final oocyte maturation**

In some studies, the use of GnRH-a in the final oocyte maturation has similar or better results compared to hCG trigger (7-11). Unlike hCG trigger, GnRH-a trigger stimulates FSH surge in addition to LH surge. FSH surge, in the mid-cycle, has a specific effect on oocyte maturation and leads to a further expansion of cumulus cells surrounding the oocyte and release of proteolytic enzymes involved in the process of ovulation(12-15). Lamb *et al* by adding a dose of FSH to the hCG trigger, showed better recovery of oocyte and higher fertilization rates in IVF compared with hCG trigger alone (16). Another advantage of this method is more maturity of the nucleus and the resumption of meiosis and eventually increasing the number of Metaphase II oocytes (9, 10, 17-19). In addition, increased levels of LH following injection of hCG is slower than that following GnRH-a trigger (20). Overall, GnRH-a trigger with effects of FSH along with the LH in the final follicular maturation, may result a more physiological maturity. Likely, more maturity of oocyte might be related to increase faster in LH surge compared with an increase of LH after 10.000 IU IM injection of hCG and also a concomitant increase of FSH (21).

GnRH-a decreases significantly the risk of OHSS and gradually is used in most clinics to induce final oocyte maturation in patients with the risk of OHSS (22). Although a few case of OHSS following GnRH-a trigger can be seen in the literature, in general using GnRH-a trigger, almost declines the risk of OHSS as a complication of ovarian stimulation by gonadotropins and its incidence is less common than hCG trigger (23, 24). Also, by diminishing OHSS following GnRH-a trigger, health care costs would be decreased. It seems that in the patients who are at risk of OHSS, GnRH-a trigger instead of hCG trigger provides an opportunity to continue the cycle and fresh embryo transfer (20). In the past, this protocol was followed to freeze all embryos in many cases. Recent modifications of luteal phase after GnRH-a trigger make it possible to transfer embryo in the same cycle for many women at the risk of OHSS and provide a good outcome (7, 8).

In addition, reduction of immature oocyte syndrome is as a result of GnRH-a trigger (25). Immature oocyte syndrome is defined as a situation that more than 25% of oocyte retrieval after ovarian stimulation in IVF/ICSI cycles are immature despite the right prescription of hCG for triggering and accurate time of oocyte collection. Following this syndrome, there will be lower pregnancy rates with causes less known (25). In a recent study of 27 women with a prior history of the immature oocyte syndrome resulting from hCG triggering, in their next cycle the mixture of GnRH-a (leuprolide acetate, 1 mg) and hCG (5.000-10.000 IU) were used to trigger resulting to retrieve more metaphase II oocytes which was significantly higher compared to previous cycles. Consequently, the high quality embryos for transfer were obtained.

**Role of GnRH -a trigger to control OHSS**

OHSS is the most serious complication and potentially fatal caused by controlled ovarian stimulation (COS) in ART (23). The biggest cause of OHSS is the presence of hCG, so that in early OHSS, the cause is exogenous hCG while delayed OHSS is often due to production of endogenous hCG following the pregnancy. HCG and LH activate the LH receptors, although the half-life of LH is less than 60 minutes, while hCG half-life is more than 24 hours (26). The long half-life and sustainable luteotrophic activity of hCG raise significantly vascular permeability stimulated by vascular endothelial growth factor (VEGF) as the major vascular mediator of OHSS (27). In order to decline the risk of OHSS, several strategies have been introduced, such as coasting technique, in vitro maturation (IVM), and finally GnRH-a triggering. In coasting technique, stopping the ovarian stimulation lead to dropping estrogen levels and induced atresia in the smaller follicles to reduce the incidence of OHSS. Unfortunately, this method has medium effect on the incidence of OHSS, and fewer oocytes often grew compared to those without coasting or even compared with GnRH-a trigger (28, 29).

Another method to prevent OHSS is oocyte collection while most follicles are still small. This method, named IVM, can increase the number of immature oocytes (30). At present, researchers propose to use GnRH-a trigger instead of hCG trigger in patients of IVF/ICSI cycle with antagonist protocol and at high risk of OHSS, thus the possibility of achieving a greater number of mature oocytes from patients of high responder with low risk of OHSS is provided (29). 

The most main clinical advantage of GnRH-a trigger is a potential to induce a rapid and reversible luteolysis and therefore decreasing the risk of OHSS progression (31). On the other hand, this is concomitant with severe luteal phase defect resulting from a short period of the induced LH and FSH peak. besides, it particularly inhibits the secretion of vasoactive products, especially VEGF, from the corpus luteum (32).

Recent studies have shown that gonadotropin and steroid levels during the luteal phase were significantly different in patients triggered by GnRH-a from hCG (33). Furthermore, the gene expression of enzymes involved in steroidogenesis, estrogen and progesterone, at the time of oocyte collection in patients with GnRH-a triggering to final oocyte maturation is less than hCG triggering (32). In addition, a significant reduction of VEGF expression in patients receiving GnRH-a is obvious that can explain the cause of prevention of early OHSS (27, 32-35). Although few cases of OHSS after GnRH-a trigger have been reported, it can be stated that trigger with GnRH-a without hCG approximately eliminates early OHSS (23, 36). 

In the largest RCT study performed up to now 266 women at the low risk of OHSS (≤14 follicles ≥11 mm) in a cycle of IVF/ICSI were divided into two groups on the trigger day; in the first group a bolus of 0.5 mg GnRH-a (buserelin) was administered while in another group 5.000 IU hCG was given (8). Women in the group triggered with GnRH-a had developed a mean of 8.1 follicles compared with 7.7 in hCG group. In the GnRH-a trigger group, following the trigger a bolus of 1.500 IU hCG in the oocyte retrieval day and another bolus of 1.500 IU hCG on oocyte retrieval +5 days to maintain the luteal phase were administered. In addition, the patients in two groups received the standard luteal phase support including oral estradiol and vaginal progesterone. In this study, although the ongoing pregnancy rate (OPR) did not differ between two groups, two cases of delayed moderate OHSS was reported in the first group.

In 2011 another study by koll *et al* was carried out on the effects of GnRH-a trigger in 15 women at low risk of OHSS with a history of previous IVF failure. The patients received one bolus of GnRH-a (triptorelin; decapeptiyle, Ferring) 0.2 mg for the final oocyte maturation and then to support the luteal phase, two boluses of 1.500 IU hCG were administrated, one day and four days after oocyte retrieval, respectively. These patients were not recieved any medication for luteal phase support. OPR was reported 47% and no cases of OHSS were found (37).

To determine whether GnRH-a trigger in women at high risk of OHSS is safe or not, a clinical trial consisted of 118 women was conducted. In this RCT, triggering was done in one group with hCG and in another group using GnRH-a. On average 14 oocytes were taken in above mentioned groups. OHSS was reported to be 3% in women with hCG trigger and no cases of OHSS were seen after GnRH-a trigger (8). Similar studies were performed by Tremellen and Radesic and lliodromiti *et al* in which a high level of OPR and low levels of abortion were reported in GnRH-a trigger group, resulting in both studies for delayed OHSS 1.4% and 0.72%, respectively (38, 39).

Considering all studies, it can be concluded that GnRH-a trigger followed by a small bolus of hCG and embryo transfer in the same cycle prevents developing OHSS in high risk women (the average of 25 follicles or less with 11 mm in diameter). With this method in high responders (with an average of 17-18 oocytes), a significant decline can be seen in expected OHSS (3). However, further studies determine the maximum number of oocytes and embryos to transfer in the same cycle will be necessary.

**GnRH -a trigger and all freezing embryos (Segmentation of cycle IVF)**

Freezing all oocytes or embryos after GnRH-a trigger and transferring in the next cycle has recently been proposed that is called segmentation of IVF cycle (40, 41). This procedure has been used with very good results in women who were exposed to risk of OHSS and also in women who needed fertility preservation (10, 40, 42). Moreover, another advantage is that the avoidance of embryo exposure to high concentrations of steroids following ovarian stimulation, which damages the endometrial receptivity and also are embryotoxic (43). Human studies have provided evidence of histologic changes in the endometrial tissue during implantation and in the development of the placenta following ovulation stimulation as well (44).

In some studies on normal responders who were at low risk of OHSS, OPR in the segmentation group was significantly higher than that when embryo transfer was done in the same cycle. Furthermore, a meta-analysis study support segmentation cycles to show that pregnancies resulting from frozen-thawed embryo transfer (FET) in IVF has better obstetric and perinatal outcomes than fresh embryo transfer (45). It is necessary to note that segmentation cycle requires very precise planning in the process of frozen-thawed embryo which is not available in all IVF centers. In addition, a number of researchers have reported a high rate of pregnancy loss, fetal abnormalities and epigenetic changes in FET cycles (46-50). At present, few studies have focused on the children resulting the FET cycles. For most patients and clinicians, embryo transfer in the same cycle leading to a healthy child is still the standard and preferred method of IVF.

**Luteal phase support following GnRH-a trigger**

As noted earlier, a bolus of hCG first induces oocyte maturation, follicle luteinization and finally causing production of endogenous progesterone for implantation. Despite the removal of large quantities of granulosa cells in the oocyte retrieval, the corpus luteum under the influence of hCG is able to release efficient progesterone in order to stimulate uterus changes for embryo implantation. So, hCG trigger has provided a simple method in the clinic for fresh embryo transfer (20). This conventional method has been modified by another trigger, GnRH-a, which unlike hCG, does not affect the early luteal phase. On the other hand, the GnRH-a trigger reduces LH levels through pituitary down-regulation, so that the amount of LH is inadequate for continuing the function of the corpus luteum (20). The reduction of the activity of the corpus luteum caused to decrease the progesterone levels in luteal phase which is very low for optimal embryo implantation (51). Therefore, the use of GnRH-a trigger without accurate luteal phase support causes a decrease in pregnancy rate. The preliminary results of the administration of GnRH-a trigger for final oocyte maturation revealed unsatisfactory results so that the high rate of pregnancy loss was associated with a significant reduction in OPR (9, 52).

In 2006, the initial meta-analysis of three RCTs reported a significant decrease in pregnancy and raise in the pregnancy loss (53). But further investigations revealed that these disappointing results was due to the utilize of standard luteal phase support following GnRH-a trigger. As well, after COS, supraphysiologic levels of estrogen and progesterone directly inhibit LH secretion from the pituitary and has a negative feedback effect on the hypothalamic-pituitary axis (54-56). 

During the luteal phase, LH performs a significant role not only in performance of corpus luteum but also in increasing the expression of growth factors and cytokines which implicated in the initial implantation (57-59). Following the failure of corpus luteum, serum levels of estrogen and progesterone significantly decrease which have adverse effects on endometrial receptivity in luteal phase (60). The mean of the luteal phase period without the use of supportive agents maybe very short after GnRH-a trigger for oocyte donors (about 4 days) in comparison with common hCG trigger (13 days) that shows a defection in corpus luteum function (61). 

Beal *et al* suggested that in GnRH-a trigger cycles, post-monitoring following oocyte pick up is crucial and if insufficient response of LH was observed, booster dose of hCG must be used (62). The least effective LH serum levels is 12-15 IU/L about 12 hours after the trigger while the most desired result is obtained when the amount is 50 IU/L (63, 64). Despite using intramuscular progesterone as supplementation in luteal phase support, the progesterone, like estradiol, had reduced levels (21, 65). An important point is to adopt strategies to advance luteal phase steroid profile that raise endometrial receptivity in order to increase the rate of live birth to an acceptable level devoid of OHSS risk progression (66).

Overall, two approaches to support luteal phase after GnRH-a trigger have become common in recent years: the European approach versus the American approach. In the European approach, the use of endogenous steroid production by the corpus luteum is done through complementary exogenous hCG and in the American approach the use of exogenous steroids with low dose adjuvant of hCG in selected cases is of interest. Both approaches have had the high fertility outcomes in patients at high risk of OHSS (8, 24, 38, 67, 68).

**American approach, Intensive LPS**

Recently, the idea of luteal phase support using just steroids after GnRH-a triggering in patients with a raised risk of OHSS has been proposed. This concept was first introduced by Babyof et al in 2006 and again in 2008 by Enegman et al (69, 70). In order to achieve the optimum level of luteal phase support, Engmann *et al* conducted a randomized clinical trial (RCT). They reported a high rate of OPR (53.3%) by monitoring of serum levels of steroids and intensive luteal phase support after GnRH-a trigger. In this way, the women were administered daily 50 mg IM progesterone beginning after oocyte collection to 10th week of pregnancy, and also since the next day of oocyte collection they received three 0.1 mg estradiol patches every other day. 

At three different times (the day of embryo transfer, a week later oocyte collection and weekly after that) serum levels of estrogen and progesterone were evaluated. In order to keep serum estrogen levels over 200 pg/ml, either the dose of the patches, up to four patches (0.1 mg) in every day or by adding the oral micronized estrogen was done. Also serum level of progesterone was retained more than 20 ng/ml using IM progesterone dose up to a maximum 75 mg daily or by adding micronized vaginal progesterone (24).

The appropriate method of progesterone prescription in ovarian stimulation is still suspected (71, 72). It is possible that after GnRH-a trigger, IM method is preferred due to abnormal luteal phase and its necessity for sufficient protection and monitoring at this time. Available evidence is poor to support exogenous estrogen after the hCG trigger in IVF cycle, but it may be necessary due to dysfunctional corpus luteum after GnRH-a trigger (70, 71, 73). Transdermal estradiol patches is superior in comparison with oral estradiol because of the lack of liver passage. Since, endogenous hCG may not increase sufficiently during the luteal phase and early pregnancy, following GnRH-a trigger steroid supplementation should be continued in order to avoid early leuteolysis of corpus luteum. 

Enegmann *et al* did a retrospective study in patients at OHSS risk with a maximum estrogen level peak less than 4.000 pg/ml. In order to obtain final oocyte mturation, patients received dual trigger (leuprolide acetate 1 mg +1000 IU hCG) in association with intensive luteal phase support (LPS). The results demonstrated higher implantation (41.9%, 22.1%, respectively) and live birth rates (58.8%, 36.8%, respectively) in dual trigger versus only GnRH-a trigger. In this study, it was observed if the intensive LPS was applied, with GnRH-a only, in patients with a maximum serum estradiol level of more 4.000 pg/ml, it could be an effective way to remain satisfactory levels of pregnancy. In patients with the highest serum estradiol levels less than 4000 pg/ml, dual trigger (GnRH-a and 1.000 IU hCG) may be appropriate to achieve the favorable rate of pregnancy by avoiding of OHSS. At present, the perfect type for luteal phase support after GnRH-a trigger is unknown, but some evidences indicate the importance of severe steroid support and serum monitoring through steroid dose regulation.

**European** **approach** **(GnRH-a in combination with** **hCG supplement)**

In European protocol, Humaidan *et al* performed first a pilot RCT conducted on 45 patients (at low risk of OHSS) to evaluate a new method in which a single dose of hCG (1.500 IU) was given 12-35 hrs after GnRH-a trigger, followed by standard methods of LPS. Patients were divided into three groups. In first group GnRH-a trigger with hCG 12 hrs later, in second group GnRH-a rigger with hCG 35 hours later, and in the third group hCG trigger (10.000 IU) were performed. Although, adequate early luteal phase support was seen in GnRH-a trigger groups by adding 1500 IU hCG (12/35 h) after the trigger, but the mid-luteal phase progesterone levels in the second group was higher than the first group.

Meanwhile, a significant difference in clinical pregnancy rate (CPR) between 35-hr group and the hCG trigger group was not observed while the pregnancy rate was significantly lower in the group of 12-hr. So, they concluded that the perfect time of 1500 IU hCG after GnRH-a trigger seems to be 35 hours after the trigger. Complete separation of ovulation trigger from luteal phase support was mentioned another benefit of the 35-hour in this study (74). An RCT including 302 patients with normal gonadotropin levels divided into two groups, hCG trigger or GnRH-a trigger with a bolus of 1.500 IU hCG after 35 hours ,there was not a significant difference in birth rates between two groups (24% vs. 31%, respectively) and the incidence of OHSS rate (moderate and severe), was 2% after hCG trigger and no OHSS case in GnRH-a trigger was reported (7).

In this study, there was 7% difference between the two groups in the rate of delivery. Humaidan *et al* conducted an RCT in 390 patients for comparison between GnRH-a trigger and hCG trigger where the hCG dose was regulated regarding the ovarian response during the stimulation. This means that the patients with 14 or fewer follicles with size greater than 11 mm (low risk for OHSS) received two bolus of hCG, 1500 IU hCG on the day of oocyte retrieval +5 days in addition to 1500 IU on oocyte retrieval day. In patients with 15-25 follicles larger than 11 mm single bolus 1500 IU hCG 35 hours after the GnRH-a trigger was administered. All patients also received a standard level of luteal phase support. Patients with more than 25 follicles were excluded from the study. The results showed no significant difference in the CPR between two groups, but the superiority was in favor of GnRH-a trigger. 3% incidence of OHSS was reported in patients with a high risk of OHSS in hCG trigger (8). However, the addition of hCG for luteal phase support after GnRH-a trigger in patients with high response significantly increased risk of delayed OHSS (75).

Suitable time for administration of low dose of hCG (12 vs. 35 hrs) after GnRH-a trigger is a subject of debate. Humaidan *et al* showed when the hCG was given 12 hours after GnRH-a trigger, the mid-luteal phase progesterone concentration and pregnancy outcome were poor (74). It seems that a period of resistance in early corpus luteum leads to impair the response of luteinizing granulosa cells against exogenous hCG stimulation. Dual trigger with GnRH-a and low dose of hCG have potential effect on oocyte maturation while low doses of hCG on the day of oocyte removal can affect the corpus luteum function and endometrial receptivity. Now, further studies are necessary (66).

**Luteal phase support** **with** **recombinant LH**

The results of low-dose hCG proved the hypothesis that luteal phase defect can be modified by adding exogenic LH. Papanikolaou *et al* suggested to support the luteal phase with recombinant LH in combination with progesterone to overcome endocrine deficiencies of luteal phase and endometrium and repair the potential for implantation after GnRH-a triggering (76). In a prospective randomized study, 17 patients in control group received 250 µg rhCG as the trigger and standard luteal phase support was performed by vaginal progesterone. In another group, 18 patients received 0.2 mg triptorlin to trigger and standard luteal phase support was done by progesterone in combination with rLH (6 doses every 2 days of 300 IU). The results showed that implantation rate were similar in both groups and no cases of OHSS were reported (76).

**Luteal phase support without exogenous progesterone in IVF**

This method emphasizes the role of endogenous progesterone following administration of the hCG exogenous in GnRH-a trigger which was considered first time in a pilot study. In this study, 15 normoresponders with antral follicle count (AFC) between 5-12, who had previous IVF failure, received GnRH-a trigger with 1500 IU hCG on the day of oocyte retrieval and also oocyte retrieval +3 days without any other supplementation. The OPR was 47%, and no case of OHSS was observed (37).

For more investigations of luteal phase support without exogenous progesterone based on hCG a pilot RCT was designed on 90 normoresponder patients undergoing IVF that women were triggered with either hCG or GnRH-a. In the GnRH-a trigger group, women were given a small daily bolus of subcutaneous rhCG (125 IU) to support luteal phase for 14 days (the day of the pregnancy test). In contrast, another group received standard luteal phase support throughout the same duration. The results showed OPR was 42% and 39% for GnRH-a and hCG trigger, respectively (3).

A recent large clinical trial with good results of fertility following exogenous hCG for luteal phase support, was carried out on 93 normal responders (less than or equal to 14 follicles). Patients were divided into two groups of GnRH-a trigger followed by repeated doses of 125 IU daily from the day of oocyte retrieval to pregnancy test day, or hCG trigger with conventional luteal phase support including micronized vaginal progesterone. OPR was 38% in GnRH-a trigger group and 41% for hCG group. HCG without exogenous progesterone for luteal phase support after GnRH-a trigger has become a new method. This method should not be used after hCG trigger protocol due to increased risk of OHSS (66).

**Other applications of** **GnRH-a** **trigger**

**GnRH-a** **trigger in the oocyte donors**

It is thought that ovarian stimulation with GnRH antagonist protocol which is followed by GnRH-a trigger is a suitable protocol for donors(26). All oocyte donors are in the risk of early OHSS. Oocyte donors are often young women with good ovarian reserve to produce a large number of oocytes, so the possibility of OHSS in these isn’t estimated less than usual. The advantages of using GnRH-a trigger in these patients include a reduction in ovarian volume, lower abdominal distension and a short duration of the luteal phase(70, 77, 78).

**GnRH-a triggering in women with breast cancer under fertility preservation treatment**

In the first retrospective analysis on women with breast cancer to preserve fertility before chemotherapy, women were treated at one IVF cycle with aromatase inhibitor and exogenous gonadotropin. Comparison between GnRH-a trigger (27 patients) and hCG trigger (47 patients) was performed. The results showed that GnRH-a trigger caused a significant reduction in the level of estradiol during luteal phase compared to hCG trigger and also increased the number of metaphase II oocytes and further development of more embryos (10). Based on reducing the risk of OHSS and improving the result of the cycle, researchers suggested using GnRH-a trigger in all women with breast cancer undergo fertility preservation treatment using GnRH-a trigger in COS with random start protocol. in this protocol, the pituitary is capable to respond sufficiently to the GnRH-a trigger during the luteal phase (79, 80). This advantage makes possible treatment protocols for women who need to quickly freeze the oocytes before cancer treatment.

## Conclusion

Review articles have shown that the outcome of fertility in GnRH-a trigger is similar to those after the hCG trigger. So, it has been proposed that GnRH-a trigger is a convenient way for patients at risk of OHSS and also oocyte donors. Despite the advantages of using hCG for LPS, currently, articles have shown that luteal phase support with low dose of hCG cannot completely eliminate the risk of OHSS. Since early OHSS may occur even after GnRH-a trigger and as well prescription of hCG 1500 IU (hCG rescue) and the risk of delayed OHSS will be remaining during pregnancy, especially in patients at risk OHSS. Also intensive LPS method could not be effective in all patients with luteal phase deficiency, in despite of acceptable outcomes. At present the most appropriate method for LPS after GnRH-a triggering is unknown and further studies are needed.
